# Electroacupuncture Treatment Alleviates Central Poststroke Pain by Inhibiting Brain Neuronal Apoptosis and Aberrant Astrocyte Activation

**DOI:** 10.1155/2016/1437148

**Published:** 2016-09-27

**Authors:** Gui-Hua Tian, Shan-Shan Tao, Man-Tang Chen, Yu-Sang Li, You-Ping Li, Hong-Cai Shang, Xiao-Yi Tang, Jian-Xin Chen, He-Bin Tang

**Affiliations:** ^1^Key Laboratory of Chinese Internal Medicine of MOE, Beijing Dongzhimen Hospital, Beijing University of Chinese Medicine, Beijing 100700, China; ^2^Department of Tuina and Pain, Beijing Dongzhimen Hospital, Beijing University of Chinese Medicine, Beijing 100700, China; ^3^Chinese Evidence-Based Medicine Center, West China Hospital, Sichuan University, Sichuan 610041, China; ^4^Department of Pharmacology, School of Pharmaceutical Sciences, South-Central University for Nationalities, No. 182, Minyuan Road, Wuhan 430074, China

## Abstract

Electroacupuncture (EA) is reported to effectively relieve the central poststroke pain (CPSP). However, the underlying mechanism remains unclear. The present study investigated the detailed mechanisms of action of EA treatment at different frequencies for CPSP. A CPSP model was established with a single collagenase injection to the left ventral posterolateral nucleus of the thalamus. The EA-treated groups then received EA treatment at frequency of 2, 2/15, or 15 Hz for 30 min daily for five days. The pain-related behavioral responses, neuronal apoptosis, glial activation, and the expression of pain signal transmission-related factors (*β*-catenin, COX-2, and NK-1R) were assessed using behavioral tests, Nissl staining, TUNEL staining, and immunohistochemical staining, respectively. The low-frequency EA treatment significantly (1) reduced brain tissue damage and hematoma sizes and (2) inhibited neuronal apoptosis, thereby exerting abirritative effects. Meanwhile, the high-frequency EA treatment induced a greater inhibition of the aberrant astrocyte activation, accompanied by the downregulation of the expressions of COX-2, *β*-catenin, and subsequently NK-1R, thereby alleviating inflammation and producing strong analgesic effects. Together, these findings suggest that CPSP is closely related to pathological changes of the neocortex and hippocampus. EA treatments at different frequencies may exert abirritative effects by inhibiting brain neuronal apoptosis and aberrant astrocyte activation in the brain.

## 1. Introduction

As a type of the neuropathic pain, central poststroke pain (CPSP) is one of the most troublesome sequelae of stroke, which can be caused by a primary lesion that affects the central somatosensory system following intracerebral hemorrhagic stroke [1]. In the clinic, patients exhibit thermal and mechanical hyperalgesia, which produces a low quality of life. A demographic census shows that the incidence of CPSP in stroke patients was approximately 7.3%–10.5% [2]. However, recent studies on neuropathic pain mainly focus on the peripheral nervous system instead of the injury to the central nervous system, particularly the CPSP [3].

In Western medicine, CPSP is mainly treated with drugs, including analgesics, antidepressants, and anticonvulsants, which may produce resistance and addiction [4]. As an important part of Chinese traditional medicine, acupuncture treatment obtains consistent international affirmation for its reliable analgesic effect. Electroacupuncture (EA) is a form of acupuncture in which a small electric current is passed between pairs of acupuncture needles. There are some reports showing that EA could be a good treatment to alleviate neuropathic pain by regulating the sympathetic nerve [5] and has the advantages of simple operation, small damage, light side effect, reasonable cost, and hence easy acceptance by patients. Currently, the EA treatment for CPSP has achieved remarkable efficacy in the clinic. For example, acupuncture at “Zusanli” and “Baihui” can significantly reduce the pain threshold of CPSP patients [3]. However, while the EA treatment for CPSP may be effective, the mechanisms underlying its therapeutic effects are not fully understood.

It has been known that CPSP is related to intracerebral hemorrhage and thalamus infarction. When blood suddenly bursts into brain tissues, the hematoma physically disrupts and seriously damages the neurons and astrocytes, producing great suffering and economic burdens to the patients [6]. After destruction of brain tissue, astrocytes will be aberrantly activated and can facilitate the synthesis and release of various pain signal transduction-related mediators, such as Akt, extracellular signal regulated kinase, *β*-catenin, nuclear factor kappa B, cyclooxygenase-2 (COX-2), and neurokinin 1 receptor (NK-1R) [6, 7], which further promote local neuroinflammation. Following astrocytes activation and the initiation of neuroinflammation, the inflammatory mediators can enhance pain-related signal transmission and induce central sensitization to generate pain.

It was speculated that these pain signal transmission-related mediators may be associated with CPSP; therefore, the present study was designed to investigate the inhibitory effects of EA treatments at different frequency patterns on neuronal apoptosis and astrocyte activation in the brain using a rat CPSP model and to reveal the changes in the expression of various pain signal transmission-related mediators (COX-2, *β*-catenin, and NK-1R) in different regions of the brain after EA treatment at different frequency patterns.

## 2. Material and Methods 

Adult male Sprague-Dawley (SD) rats (250–300 g; *n* = 75) were obtained from the experimental animal center of Hubei Province. The care and use of animals and all experimental protocols (Permit number: 2011-SCUEC-AEC-001) for this study were performed according to the Guide for Animal Experimentation, South-Central University for Nationalities, and the Committee of Research Facilities for Laboratory Animal Sciences, South-Central University for Nationalities, China.

The experimental CPSP model was induced by single collagenase injection into the left ventral posterolateral nucleus of the rat thalamus, as previously reported [8]. All surgeries were performed under trichloroacetaldehyde monohydrate (450 mg/kg, i.p.) anesthesia and placed in a stereotaxic frame. Under stereotaxic guidance, collagenase (type IV; 0.025 U in 0.25 *μ*L cerebrospinal fluid; Sigma-Aldrich, St. Louis, MO, USA) or cerebrospinal fluid (0.25 *μ*L, containing 126.0 mM NaCl, 3.0 mM KCl, 1.4 mM Na_2_PO_4_, 1.0 mM MgSO_4_, 26.0 mM NaHCO_3_, 10.0 mM glucose, and 2.5 mM CaCl_2_, pH 7.4) was injected into the left ventral posterolateral nucleus of the thalamus (A: −3.8 mm; L: 3.3 mm from bregma; and V: 6.0 mm). Sham was injected with an equal volume of the cerebrospinal fluid vehicle. The needle was left in place for 5 min to allow for diffusion of collagenase away from the injection site and then withdrawn gently. All SD rats were randomly and double-blindly divided into seven groups: a control group (intragastric administration of saline; *n* = 15), a sham group (intragastric administration of saline after the injection of cerebrospinal fluid; *n* = 5), a model group (intragastric administration of saline after the injection of collagenase; *n* = 15), a fluoxetine group (intragastric administration of fluoxetine after the injection of collagenase; 5 mL/kg, 0.4 mg/mL; *n* = 10; a positive drug to treat the stroke through improving infarct volumes and neurobehavioral, Patheon France, Bourgoin-Jallieu, France), and EA-treated groups (2, 15, and 2/15 Hz groups; *n* = 10 for each group).

Rats were loosely immobilized on a wood plate and two stainless steel acupuncture needles were inserted into two acupoints ST36 and GV20 with a depth of 5 mm. In the EA treatment groups, the rats received EA administration on the left “Zusanli” (ST36, 5 mm lateral to the anterior tubercle of tibia) and “Baihui” (GV20, located at the midmost point between the bilateral parietal bones, forward insertion) once every day, starting from 24 h after the termination of collagenase injection for 5 days. EA (1 mA) was administered at different frequencies (2, 15, or 2/15 Hz) for 30 min every day. The EA's current was delivered with a modified current-constant Han's Acupuncture Point Nerve Stimulator (HANA-100A, Huawei Co., Ltd., Beijing, China) where the needles were connected with the electrical stimulation [9].

Hyperalgesia to thermal stimulation was measured using a plantar test (Model 37370, Ugo Basile, Varese, Italy) according to a previously described method [10]. Subsequent readings of the same paw were carried out at 0, 1, 3, and 5 day(s) after the operation. The process was repeated three times, and the mean values were taken as the threshold values.

Hyperalgesia to pressure stimulation was measured in the hindpaws of lightly restrained alert rats using a handheld measuring instrument (HR/SLY-HFM/402359, Beijing, China) referred to in a previously described method [10]. The forces required for mechanical hyperalgesia were displayed on the instrument, when the rats withdrew the paws after the stimulation. The process was repeated three times, and the mean values were taken as the threshold values.

Hyperalgesia to cold stimulation was measured with an ice-cold metal aluminum platform according to a previously described method [10]. The latency before the first response (licking, paw movements, and little leaps) to cold stimulation was recorded with a cut-off time of 60 s. The process was repeated three times, and the mean values were taken as the threshold values.

To examine the brain neuronal cell damage of CPSP rats, the rats were sacrificed via intracardial perfusion on the 5th or 7th day after the operation, and their brains were dissected and rapidly fixed for 24 h at 22°C with 4% paraformaldehyde (Sigma). The 4 *μ*m brain sections were washed by a series of grade ethanol for 5 min each time and incubated in Nissl staining solutions (Beyotime Institute of Biotechnology, Nantong, China) for 30 min at room temperature according to the manufacturer's instructions. The brain neuronal cell apoptosis was analyzed using a Nuance Multispectral Imaging System (Cambridge Research and Instrumentation Inc., Woburn, MA, USA) with instructions followed closely [11].

To examine the expressions of several pain signal transmission-related factors in CPSP rats, the 4 *μ*m brain sections were incubated overnight at 4°C with rabbit anti-COX-2 antibody (1 : 200 dilution; Cayman Chemical, Ann Arbor, MI), rabbit anti-glial fibrillary acidic protein (GFAP) antibody (1 : 400 dilution; Sigma Chemical Co., St. Louis, MO, USA), rabbit anti-*β*-catenin (1 : 400 dilution, Cayman), or rabbit-anti-NK-1R antibody (1 : 2000 dilution, Sigma) according to the manufacturer's instructions (Histofine Simple Stain Rat MAX-PO (MULTI) kit; Nichirei, Tokyo) [12].

To examine the apoptosis in CPSP rat brain sections, the 4 *μ*m brain sections were incubated with TdT-mediated dUTP Nick-End Labeling (TUNEL) reaction mixture containing biotin-labeled dUTPs at 37°C for 45 min according to the manufacturer's instructions (Boster Biochemical Techniques Co., Ltd., Wuhan, China). In a negative control, the TUNEL reaction mixture was replaced with PBS. Sections of the positive control sections were pretreated with DNase I for 10 min followed by TUNEL staining [11].

Finally, multispectral imaging analysis of all slides in each experiment was performed by using an Eclipse Ti microscope (Nikon, Tokyo) with a Nuance Multispectral Imaging System (Cambridge Research and Instrumentation Inc., Woburn, MA, USA) according to a previously described method [12].

On the 5th day, rats were sacrificed via decollation. The cortex of the damage area and the whole hippocampus tissues were rapidly dissected out and then put into the liquid nitrogen and stored in −80°C. Then the tissues were homogenized in ice-cold cell lysis buffers (Beyotime Institute of Biotechnology, Haimen, Jiangsu, China) containing 1% phenylmethanesulfonylfluoride (Beyotime Institute of Biotechnology) and 1% phosphatase inhibitor cocktail (Roche Diagnostics GmbH, Mannheim, Germany), respectively. The protein concentration of each fraction was determined using a Lowry protein assay. Equal amounts of protein were separated and transferred to polyvinylidene fluoride membranes. Then, the membranes were incubated with primary antibodies: rabbit anti-NK-1R antibody (1 : 3000 dilution, Sigma), rabbit anti-*β*-catenin antibody (1 : 500 dilution, Cayman), rabbit anti-GFAP-antibody (1 : 2000 dilution, Sigma), rabbit anti-COX-2 antibody (1 : 200 dilution, Cayman), and rabbit anti-*β*-tubulin antibody (1 : 5000 dilution; ABclonal Biotech Co., Ltd., Baltimore Avenue, USA) overnight at 4°C. After reaction with a horseradish peroxidase-conjugated anti-rabbit secondary antibody (1 : 2000 dilution; Cell Signaling Technology, Beverly, MA), the proteins were detected using an ECL detection reagent according to a previously described method [13].

The results are shown as the means ± SEM. The statistical analysis was performed by one- or two-way ANOVA, as indicated in the text, using InStat software (GraphPad Prism 5, USA). A *p* value less than 0.05 was considered to be statistically significant.

## 3. Results

**Figure 1:**
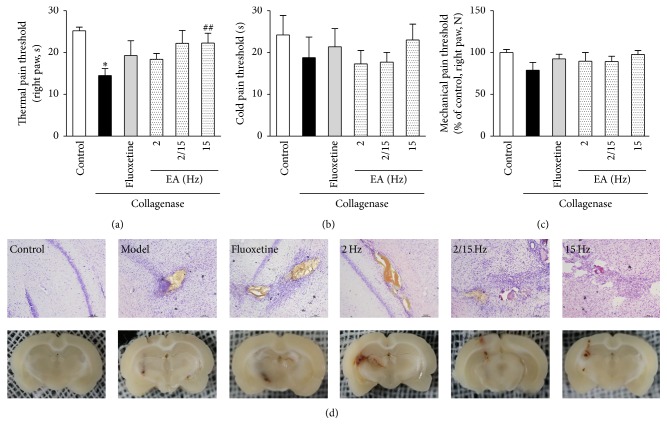
Effects of the different frequency EA treatments on pain-related behavioral responses and neuropathological changes in the brains of the CPSP rats. Changes in the thermal (a), cold (b), and mechanical (c) hyperalgesia in the contralateral paw of CPSP rats that had been treated with or without EA at different frequencies. (d) Representative photomicrographs of Nissl staining in equivalent coronal sections of the brains of the CPSP rats on the 5th day after the operation. *∗* denotes* p* < 0.05 versus the control; ## denotes* p* < 0.01 versus the model (*n* = 5 in each group; one-way analysis of variance, followed by the Newman-Keuls* post hoc* test).

As shown in Figure 1(a) and Figure I (online-only Supplementary Material available at http://dx.doi.org/10.1155/2016/1437148), on the 5th day, there was a significant difference in the thermal pain threshold between the model group (14.5 ± 1.7 s) and the control group (25.2 ± 0.9 s). The thermal pain thresholds of the rats in the fluoxetine-treated and different frequency EA-treated groups were increased (19.3 ± 3.5 s for fluoxetine; 18.4 ± 1.4 s, 22.2 ± 3.1 s, and 22.3 ± 2.3 s for 2, 2/15, and 15 Hz, resp.) compared to the rats in the model group, which also showed that the efficacy of the EA treatment, particularly the high-frequency EA treatment, was better than fluoxetine. Similar to the changes in the thermal pain thresholds, there appeared to be a decreasing trend in the cold pain threshold after the EA treatment (Figure 1(b)). However, regarding the mechanical pain threshold, the efficacies of fluoxetine and the different frequencies of EA treatment were nearly identical (92.4 ± 5.6 s for fluoxetine; 89.6 ± 10.5%, 89.2 ± 6.3%, and 97.6 ± 4.8% of the control for 2, 2/15, and 15 Hz, resp.; Figure 1(c)). Taken together, the results shown in Figures 1(a), 1(b), and 1(c) indicated that fluoxetine and EA treatment could relieve CPSP, while the administration of high-frequency EA led to the best performance.

Regarding the neuropathological changes in the brain, on the 5th day after the collagenase injection into the thalamus, the brains exhibited obvious cerebral hemorrhages, neuronal cell damage, and aberrant glial cell activation compared to those in the control group. After the treatment with fluoxetine or different patterns of EA, increased Nissl body staining, decreased hematoma sizes, and relieved inflammation were observed, which indicated that the EA treatment for CPSP was effective (Figure 1(d)). It is also important to note that the changes on the above parameters made by EA treatments exceed fluoxetine, demonstrating a better efficacy of EA treatments. Moreover, among EA treatments, the low-frequency one exhibited the best efficacy in improving the neuropathological changes in the brain.

**Figure 2:**
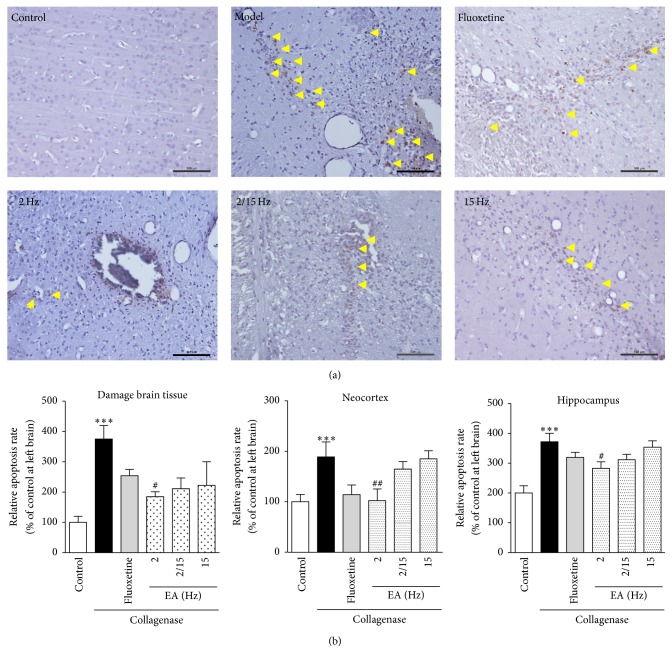
Effects of the different frequency EA treatments on neuronal apoptosis in the brains of the CPSP rats. (a) Representative photomicrographs of TUNEL staining in the brains of the CPSP rats that had been treated with or without EA on the 5th day after the operation. The yellow arrows denote neuronal cell apoptosis. (b) Determination of neuronal cell apoptosis rate in the brains of the CPSP rats (ipsilateral brain section) using a multispectral analysis. *∗∗∗* denotes *p* < 0.001 versus the control; # and ## denote *p* < 0.05 and 0.01 versus the model (*n* = 5 in each group; one-way analysis of variance, followed by the Newman-Keuls* post hoc* test).

To further analyze the correlations between EA administration and the cellular apoptosis in brain tissues, a TUNEL assay and a multispectral imaging analysis were performed to quantify the relative rates of cellular apoptosis. As shown in Figure 2(a), glial cell apoptosis was noticeable around the damaged area in the model group (375.3 ± 44.3% of the control) compared to the control group (100.0 ± 19.9%). After EA treatment at the corresponding frequencies, apoptosis was downregulated (184.4 ± 16.6%, 210.8 ± 35.7%, and 222.0 ± 78.2% of the control for 2, 2/15, and 15 Hz, resp.), and the efficacy was better than that of the fluoxetine group (254.1 ± 20.9% of the control).

As shown in Figure 2(b), apoptosis was observed in the neocortex and hippocampus sections from the model group (188.8 ± 29.9% and 185.9 ± 14.5% of the control, resp.). After the EA treatment, the relative cellular apoptosis rate in both the neocortex and hippocampus of the CPSP rats sharply decreased and the low-frequency EA treatment exhibited the best efficacy (102.3 ± 23.1% of the control and 141.4 ± 10.9% of the control, resp.), which was closely followed by the fluoxetine group (114.2 ± 19.2% and 159.8 ± 8.4% of the control, resp.).

**Figure 3:**
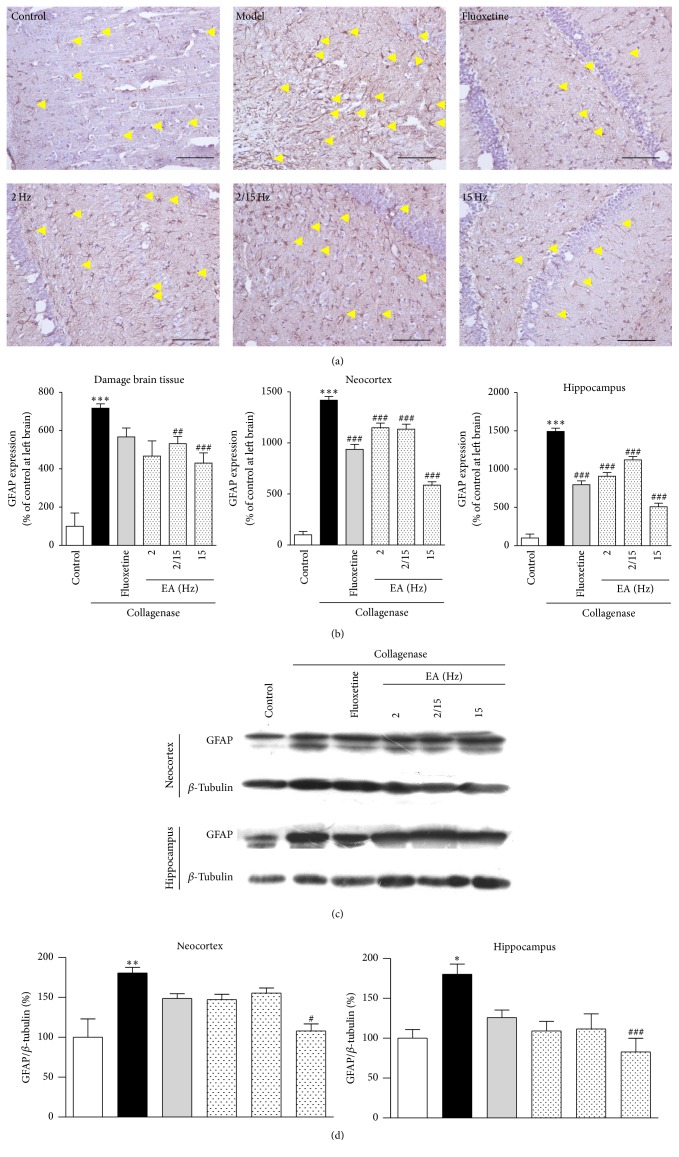
Effects of the different frequency EA treatments on GFAP expression in the brains of the CPSP rats. (a) Representative photomicrographs of immunohistochemical staining of GFAP expression in the brains of the CPSP rats that had been treated with or without EA on the 5th day after the operation. The yellow arrows denote glia cells. (b) Determination of GFAP expression in the brains of the CPSP rats using a multispectral analysis (ipsilateral brain section). (c) Representative Western blots of ipsilateral GFAP expression in the neocortex and hippocampus of the CPSP rats. (d) Western blot determination of (c). *∗*, *∗∗*, and *∗∗∗* denote *p* < 0.05, 0.01, and 0.001 versus the control; #, ##, and ### denote *p* < 0.05, 0.01, and 0.001 versus the model (*n* = 5 in each group; one-way analysis of variance, followed by the Newman-Keuls* post hoc* test), respectively.

As shown in Figure 3(a), the astrocytes in the model group were hypertrophic and showed a remarkable increase in the number of cells. On the contrary, there were fewer and smaller astrocytes in the fluoxetine- and EA-treated groups.

In Figure 3(b), the expression of GFAP in the model group (716.9 ± 22.6% of the control) was significantly increased after the collagenase injection compared to the control group (100.0 ± 34.8%). After the EA treatment, GFAP expression (466.9 ± 75.6%, 531.7 ± 38.0%, and 430.5 ± 52.9% of the control for 2, 2/15, and 15 Hz, resp.) decreased, and the high-frequency EA was more effective. In addition, the efficacy of the fluoxetine group (567.3 ± 46.0% of the control) was marginally reduced compared to the EA-treated groups.

Aberrant GFAP expression was observed in the neocortex and hippocampal sections from the model group (1419.4 ± 36.3% and 1491.7 ± 43.8% of the control, resp.). After the EA treatment, GFAP expression was sharply decreased, and the high-frequency EA treatment exhibited highest efficiency (586.4 ± 33.2% and 509.0 ± 47.2% of the control, resp.). Moreover, the efficacy of the fluoxetine group (938.2 ± 48.3% and 797.8 ± 50.1% of the control, resp.) was marginally reduced compared to the EA-treated groups.

To further confirm the inhibitory effect of the EA treatment on GFAP expression, Western blotting analysis was performed using the proteins extracted from the neocortex and hippocampus of the rats in the same groups (Figure 3(c)). GFAP expression was also significantly increased in the neocortex and hippocampus from the rats in the model group (180.6 ± 7.1% and 180.3 ± 12.6% of the control, resp.). After the EA treatment, the GFAP expression levels in the neocortex and hippocampus were decreased (107.9 ± 8.8% and 82.7 ± 17.1% of the control, resp.), and the high-frequency EA treatment exhibited better efficacy. In addition, GFAP expression in the tissues from the fluoxetine group (148.8 ± 5.7% and 125.9 ± 9.2% of the control, resp.; Figure 3(d)) was increased compared to those in the EA groups. As a result, the EA treatment, particularly the high-frequency EA treatment, attenuates aberrant glial activation, thereby reducing neuronal cell damage.

**Figure 4:**
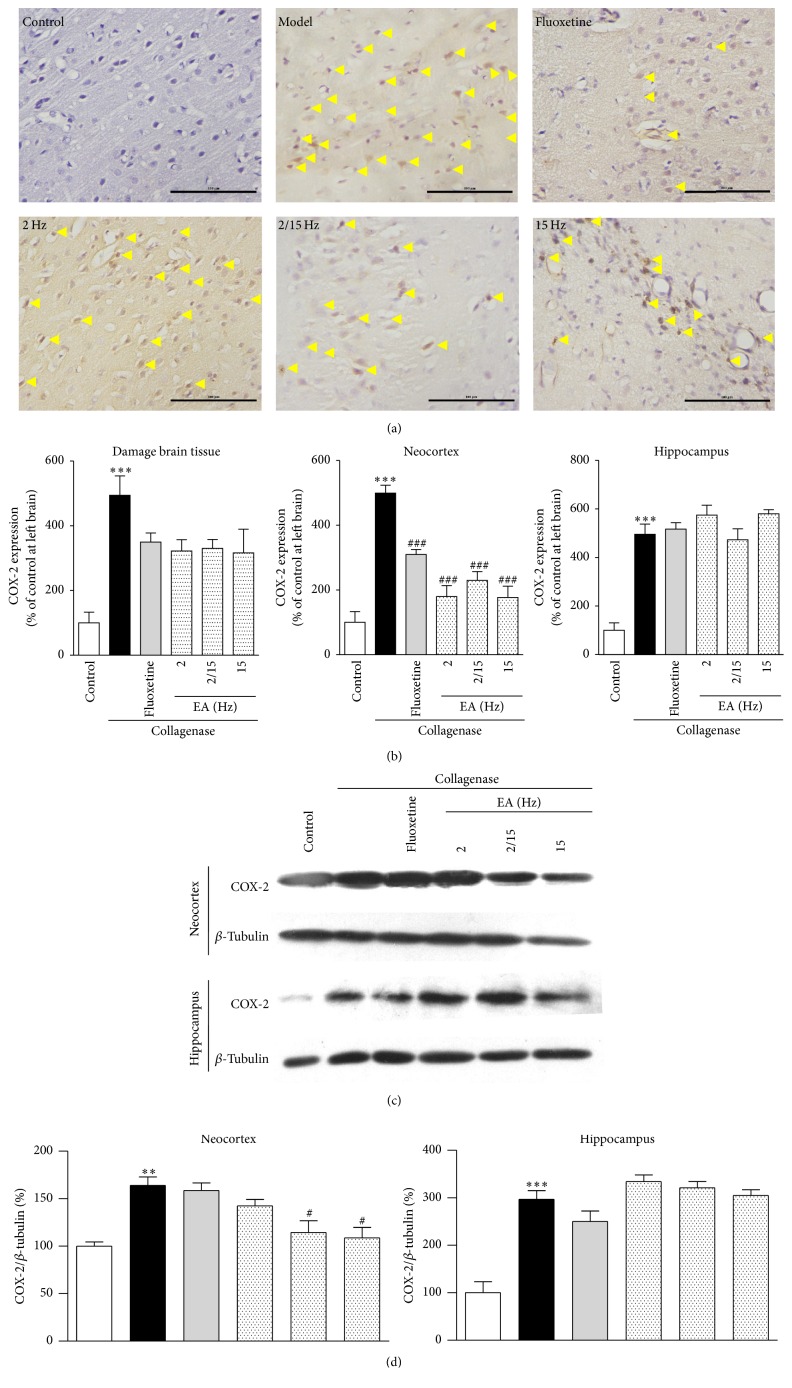
Effects of the different frequency EA treatments on COX-2 expression in the brains of the CPSP rats. (a) Representative photomicrographs of immunohistochemical staining of COX-2 expression in the brains of the CPSP rats that had been treated with or without EA on the 5th day after the operation. (b) Determination of COX-2 expression in the brains of the CPSP rats using a multispectral analysis (ipsilateral brain section). (c) Representative Western blots of COX-2 expression in the ipsilateral neocortex and hippocampus of the CPSP rats. (d) Western blot determination of (c). *∗∗* and *∗∗∗* denote *p* < 0.01, 0.001 versus the control; # and ### denote *p* < 0.05, 0.001 versus the model (*n* = 5 in each group; one-way analysis of variance, followed by the Newman-Keuls* post hoc* test).

As shown in Figure 4(a), there were a massive inflammatory infiltration and a large number of COX-2-positive cells around the damaged brain tissue after the collagenase injection compared to the control group. After an intragastric administration of fluoxetine or the EA treatment, a reduced number of COX-2-positive cells were observed in brain tissues, and the inflammation was relatively reduced compared to the control group, which indicated that the EA treatments with different frequency patterns were effective at alleviating CPSP. We further quantified COX-2 expression using a multispectral quantitative technology. COX-2 expression (322.0 ± 35.0%, 330.3 ± 27.5%, and 316.2 ± 73.6% of the control for 2, 2/15, and 15 Hz, resp.) decreased after the EA treatment compared to the model group (494.0 ± 60.4% of the control) and was slightly better than the fluoxetine group (349.7 ± 27.9% of the control).

Moreover, we quantified COX-2 expression in the the neocortex and hippocampus. The EA treatment decreased COX-2 expression in the neocortex (179.5 ± 34.1%, 229.8 ± 26.8%, and 176.5 ± 35.1% of the control for 2, 2/15, and 15 Hz, resp.) compared to that in the model group (499.2 ± 24.3% of the control), and the efficacy of the high-frequency EA treatment was better than the EA treatments of other patterns and fluoxetine (309.7 ± 15.4% of the control). However, the phenomenon was not evident in the hippocampus (Figure 4(b)).

As shown in Figure 4(c), we further investigated the inhibitory effects of the EA treatment on COX-2 expression by Western blotting analysis using the proteins extracted from the neocortex and hippocampus of the rats in the same groups. As expected, the EA treatments of different patterns and fluoxetine significantly decreased the COX-2 expression levels in the neocortex (142.4 ± 6.7%, 114.4 ± 12.3%, and 108.7 ± 19.8% of the control for 2, 2/15, and 15 Hz, resp.; 158.5 ± 8.1% of the control for fluoxetine) compared to the model group (163.9 ± 8.9% of the control), and the high-frequency EA treatment exhibited better efficacy (Figure 4(d)). Therefore, we could conclude that the EA treatment, particularly the high-frequency EA treatment, could inhibit COX-2 expression to attenuate the inflammation induced by the collagenase injection.

**Figure 5:**
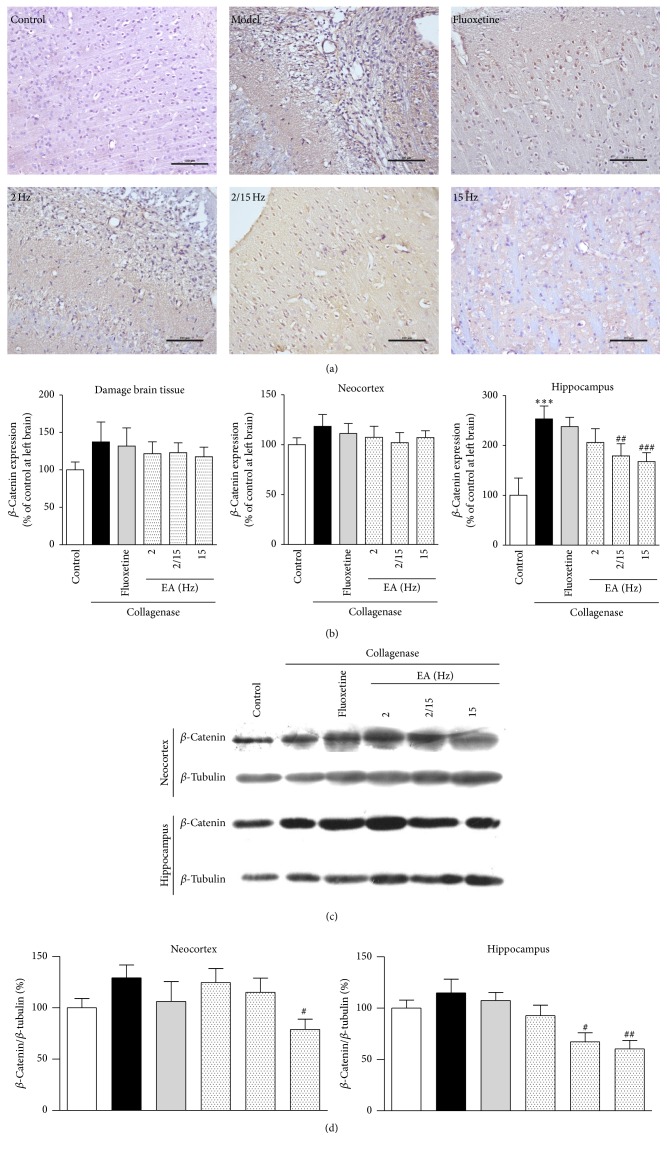
Effects of the different frequency EA treatments on *β*-catenin expression in the brains of the CPSP rats. (a) Representative photomicrographs of immunohistochemical staining of *β*-catenin in the brains of the CPSP rats that had been treated with or without EA on the 5th day after the operation. (b) Determination of *β*-catenin expression in the brains of the CPSP rats brain using a multispectral analysis (ipsilateral brain section). (c) Representative Western blots of *β*-catenin expression in the ipsilateral neocortex and hippocampus of the rats. (d) Western blot determination of (c). *∗∗∗* denotes *p* < 0.001 versus the control; #, ##, and ### denote *p* < 0.05, 0.01, and 0.001 versus the model (*n* = 5 in each group; one-way analysis of variance, followed by the Newman-Keuls* post hoc* test).

Next, the effects of the EA treatment on the expression of *β*-catenin in the CPSP rats were investigated. As shown in Figure 5(a), a large number of *β*-catenin-positive cells were observed around the damaged brain tissues, particularly in the cytoplasm and cell nucleus of the CPSP rats compared to the control group. After the EA treatment, the number of *β*-catenin-positive cells was relatively reduced compared to that in the control or fluoxetine groups, which indicated that the EA treatment was effective on CPSP. We further quantified *β*-catenin expression using a multispectral quantitative technology. As shown in Figure 5(b), *β*-catenin expression (121.7 ± 15.8%, 123.0 ± 13.1%, and 117.5 ± 12.9% of the control for 2, 2/15, and 15 Hz, resp.) decreased after the EA treatment compared to the model group (137.4 ± 26.7% of the control) and the fluoxetine group (131.8 ± 24.2% of the control), confirming that the high-frequency EA treatment exhibited better efficacy.

Moreover, *β*-catenin expressions in the different brain regions, such as the neocortex and hippocampus, were quantified and compared. Compared to the model group (252.9 ± 26.2% of the control), the high-frequency EA treatment (167.8 ± 17.6% of the control) inhibited *β*-catenin expression in the hippocampus of the CPSP rats, and the high-frequency EA treatment exhibited better efficacy (Figure 5(b)).

To further study *β*-catenin expression after the EA treatment, we evaluated the efficacy of EA treatment by Western blotting analysis using the proteins extracted from the neocortex and hippocampus of the rats from the same groups (Figure 5(c)). *β*-Catenin expression decreased in the neocortex and hippocampus of the 15 Hz group (neocortex: 78.9 ± 10.0%; hippocampus: 60.4 ± 8.2% of the control) after the EA treatment and the expression was further reduced in the fluoxetine group (neocortex: 106.2 ± 19.4% of the control; hippocampus: 107.4 ± 7.9% of the control) compared to the model group (neocortex: 129.2 ± 12.4% of the control; hippocampus: 114.8 ± 13.3% of the control). Moreover, the high-frequency EA treatment exhibited better efficacy (Figure 5(d)). Consequently, the high-frequency EA treatment inhibited *β*-catenin expression and produced analgesic effects.

**Figure 6:**
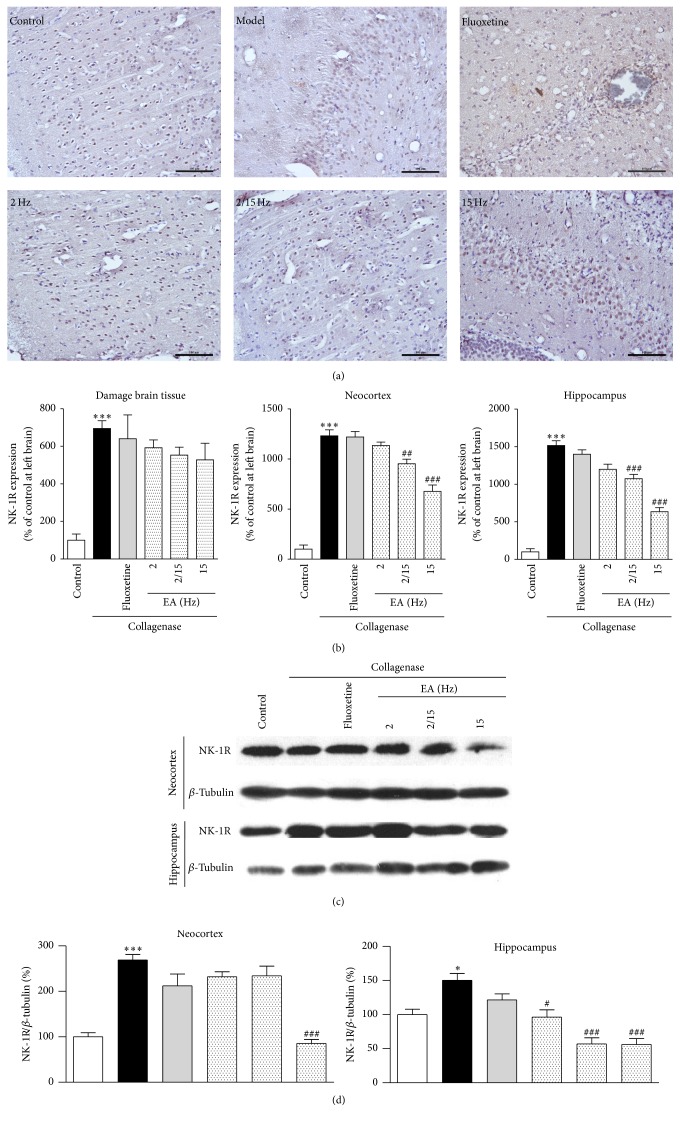
Effects of the different frequency EA treatments on NK-1R expression in the brains of the CPSP rats. (a) Representative photomicrographs of immunohistochemical staining of NK-1R expression in the brains of the CPSP rats that had been treated with or without EA on the 5th day after the operation. (b) Determination of NK-1R expression in the brains of the CPSP rats using a multispectral analysis (ipsilateral brain section). (c) Representative Western blots of NK-1R expression in the ipsilateral neocortex and hippocampus of the CPSP rats. (d) Western blot determination of (c). *∗* and *∗∗∗* denote *p* < 0.05 and 0.001 versus the control; #, ##, and ### denote *p* < 0.05, 0.01, and 0.001 versus the model (*n* = 5 in each group; one-way analysis of variance, followed by the Newman-Keuls* post hoc* test).

As shown in Figure 6(a), a large number of NK-1R-positive cells were observed around the damaged brain tissues after the collagenase injection compared to the control groups, particularly the model group. However, a smaller number of NK-1R-positive cells were observed in the fluoxetine group compared to the model group. After the EA treatment, the number of NK-1R-positive cells was relatively reduced compared to the fluoxetine group, which indicated that the EA treatment for CPSP was effective. To further demonstrate the expression, NK-1R expression was further quantified using a multispectral quantitative technology. As shown in Figure 6(b), NK-1R expression (592.0 ± 41.9%, 553.4 ± 31.9%, and 528.5 ± 88.1% of the control for 2, 2/15, and 15 Hz, resp.) decreased after the EA treatment compared to the model group (695.2 ± 10.8% of the control). The level of NK-1R expression in the fluoxetine group (640.5 ± 97.1% of the control) was between that of the model group and that of the EA groups, which showed that the high-frequency EA treatment exhibited better efficacy.

Moreover, we quantified NK-1R expressions in different brain regions, such as the neocortex and hippocampus, respectively. NK-1R expression was significantly decreased in the neocortex and hippocampus (neocortex: 528.5 ± 88.1% of the control; hippocampus: 635.3 ± 53.2% of the control) of the EA-treated groups compared to the model group (neocortex: 1232.0 ± 60.4% of the control; hippocampus: 1516.3 ± 62.6% of the control), and the effect of fluoxetine (neocortex: 1220.6 ± 54.4% of the control; hippocampus: 1399.1 ± 59.6% of the control) was not as significant as the EA treatments. Therefore, the high-frequency EA treatment exhibited better efficacy (Figure 6(b)).

To further study NK-1R expression after the EA treatment, the efficacy of EA treatment was evaluated by Western blotting analysis using the proteins extracted from the neocortex and hippocampus of the rats in the same groups (Figure 6(c)). NK-1R expression was significantly decreased in the neocortex and hippocampus of the 15 Hz group (neocortex: 85.3 ± 8.7% of the control; hippocampus: 56.1 ± 8.6% of the control) after EA treatment compared to the model group (neocortex: 268.7 ± 12.4% of the control; hippocampus: 150.3 ± 9.8% of the control), whereas NK-1R expression in the fluoxetine group (neocortex: 212.0 ± 26.0% of control; hippocampus: 121.3 ± 8.9% of control) was higher than that of the EA groups. It was speculated that the high-frequency EA treatment exhibited better efficacy (Figure 6(d)). Hence, the high-frequency EA treatment could significantly downregulate NK-1R expression, thus exerting a direct analgesic effect.

## 4. Discussion

In the present study, we confirmed that low-frequency and high-frequency EA treatments exert analgesic effects by inhibiting neuronal cell apoptosis and aberrant astrocyte activation in the brain. Different regions of the brain, particularly the neocortex and hippocampus, are related to the generation of CPSP.

It is known that some kinds of medicine can relieve CPSP in some degree [4] and acupuncture, which has been used in China and other Asian countries for the past 3,000 years, represents a potentially valuable adjunct therapy to the existing strategies for pain relief. Some researchers have studied the process of pain and showed that neuronal cell apoptosis and abnormal astrocyte activation play an important role in pain. A growing body of evidence supports the role of astrocytes in the development of persistent pain and hypersensitivity after injury [14]. Researchers have proved that the molecular and cellular alterations in the primary sensory neurons and the neurons in the spinal dorsal horn play important roles in the pathogenesis of neuropathic pain after peripheral nerve injury [15]. However, the mechanism by which the neuronal cell apoptosis and abnormal astrocyte activation promote CPSP is not clear. As expected, we have confirmed that neuronal cell apoptosis and abnormal astrocyte activation in the brain indeed occurred in CPSP (Figures 2 and 3), and the low-frequency EA was effective at relieving neuronal cell apoptosis, while the high-frequency EA was effective at inhibiting the abnormal astrocyte activation. Moreover, the EA treatment could relieve the CPSP and its efficacy was better than that of fluoxetine (a positive drug to treat the stroke through improving infarct volumes and neurobehavioral [16]; Figure 1).

As an inducible enzyme that is pivotal in the inflammatory response, COX-2 converts arachidonic acid to prostaglandins, which are necessary for the biosynthesis and release of substance P during the development of inflammation [12]. Moreover, nerve injury evoked a positive feedback loop between COX-2 and *β*-catenin for the biosynthesis and release of substance P, which may contribute to the responses related to neuropathic pain [17]. Thus, we could speculate that the possible mechanism by which the EA treatment improved CPSP was through the downregulation of *β*-catenin expression to exert an analgesic effect.

In addition, NK-1R is an endogenous receptor for substance P. The binding of substance P to NK-1R is thought to be related to the transmission of pain signals [18]. Preclinical studies show that NK-1R antagonists exert an analgesic effect, and clinical studies in humans have shown that these antagonists are generally ineffective for the treatment of pain [19]. The upregulated NK-1R expression in the CPSP rats could be attenuated by the EA treatment (Figure 6), suggesting that the EA treatment can relieve CPSP better than NK-1R antagonists.

Moreover, some previous studies have demonstrated that the low-frequency EA treatment for CPSP inhibits neuronal cell apoptosis. Wang et al. indicated that different frequency EAs were spatially specific, and the 2 Hz EA treatment regulated more genes, which were associated with apoptosis [20]. Compared to the above observations, our present findings strongly suggested that low-frequency EA was effective at alleviating CPSP by inhibiting neuronal cell apoptosis. Xiang et al. described that the different frequencies of EA were mediated by different opioid receptors in specific areas of the central nervous system [21]; therefore, the different frequency EA treatments may have different roles in various pathways. Some other studies have proved that the analgesia induced by high-frequency EA (>100 Hz) is mainly mediated by the release of dynorphins and *κ* receptors [22]. In the present study, we have demonstrated that the high-frequency EA treatment exerted a greater analgesic effect mainly by regulating the expression of pain signal transmission-related mediators (*β*-catenin, COX-2, and NK-1R). Based on the above discussion, we are interested in exploring the therapeutic effect of EA at an alternating frequency (2/15 Hz). Unfortunately, the therapeutic effect of the 2/15 Hz EA treatment is worse than that of the 2 Hz or 15 Hz EA treatments, which will be further investigated in a future study.

Dejerine and Roussy firstly described that CPSP had a thalamic origin, which was characterized by neuropathic pain emerging from thalamic lesions, such as infarctions and bleeds [23]. The hippocampus is the most sensitive area to cerebral hemorrhage and has important roles in learning and memory, which has a close relationship with cognitive function. In addition, the prefrontal cortex is known to participate in executive function, emotions, and pain. Some studies have shown that prefrontal cortical dysfunction occurs in chronic pain patients [24]. Therefore, in our study, we investigated the expression levels of GFAP, *β*-catenin, COX-2, and NK-1R in the neocortex and hippocampus to explore their relationships with pain. Abnormal astrocyte activation has been observed in the cortex in other models of neuropathic pain [25], but not in rat CPSP model. However, GFAP expression was downregulated in the neocortex and hippocampus after the EA treatment (Figure 3). Many lines of experimental evidence emphasized the significance of blocking the COX-2 pathway in therapeutic strategies for global cerebral ischemia. COX-2 expression was dramatically increased in the injured hippocampus of animal models following global cerebral ischemia [26]. We indeed found that EA could downregulate COX-2 expression to exert an analgesic effect in the hippocampus (Figure 4). Growing evidence showed that an NK-1R antagonist could disrupt the binding of substance P to NK-1R, which significantly improved the motor and cognitive outcomes and inhibited dyskinesia in a hemi-Parkinsonian rat model [27]. Moreover, the NK-1R antagonist reduced brain edema and axonal injury in experimental models of traumatic brain injury [28]. Similar to the NK-1R antagonist, the NK-1R expression was indeed downregulated after the EA treatment (Figure 6). In a previous study, treatment with lithium ions decreased *β*-catenin expression in the ischemic cortex, when assessed 3 days after an endothelin-1 injection [29]. EA treatment downregulated *β*-catenin expression in both the cortex and hippocampus (Figure 5). Thus, we may conclude that the EA treatment for CPSP could downregulate the expression of some pain signal transmission-related mediators in different brain regions, particularly the neocortex and hippocampus.

In summary, our studies strongly confirmed that the EA treatment was effective at alleviating CPSP, and different regions of the brain, particularly the neocortex and hippocampus, have a significant relationship with the generation of pain. Low-frequency and high-frequency EA treatments may exert abirritation effects by inhibiting neuronal apoptosis and aberrant astrocyte activation. However, this finding was observed only by histomorphology and still must be verified at the protein and nucleic acid levels. Moreover, additional investigations are required to fully elucidate the signaling pathways. Nevertheless, the present findings may elucidate the mechanism of EA treatment for CPSP from a novel perspective.

## Supplementary Material

Measurement of brain neuropathological changes and pain-related behavioral responses (within 7 days) in CPSP rats.
