# Supplementary material for: Electroacupuncture Treatment Alleviates Central Poststroke Pain by Inhibiting Brain Neuronal Apoptosis and Aberrant Astrocyte Activation
Source: Neural Plast. 2016 Sep 27;2016:1437148. doi: 10.1155/2016/1437148 (PMC5059615; doi:10.1155/2016/1437148)
Supplement: Supplementary file 1 — Measurement of brain neuropathological changes and pain-related behavioral responses (within 7 days) in CPSP rats. [file 1437148.f1.doc]

**Electroacupuncture treatment alleviates central post-stroke pain by inhibiting brain neuronal apoptosis and aberrant astrocyte activation**

Gui-Hua Tian^1,3,4, #^, Shan-Shan Tao^2, #^, Man-Tang Chen^2^, Yu-Sang Li^2^, You-Ping Li^4^, Hong-Cai Shang^1^, Xiao-Yi Tang^1,3^, Jian-Xin Chen^1^, He-Bin Tang^2^,*

Sponsorships or competing interests that may be relevant to content are disclosed at the end of this article.

^1^ Key Laboratory of Chinese Internal Medicine of MOE, Beijing Dongzhimen Hospital, Beijing University of Chinese Medicine, 100700 Beijing, China (http://www.bucm.edu.cn/);

^2^ Department of Pharmacology, School of Pharmaceutical Sciences, South-Central University for Nationalities, No. 182, Minyuan Road, 430074 Wuhan, China (http://www.scuec.edu.cn/);

^3^ Department of Tuina and Pain, Beijing Dongzhimen Hospital, Beijing University of Chinese Medicine, 100700 Beijing, China (http://www.bucm.edu.cn/);

^4^ Chinese Evidence-based Medicine Center, West China Hospital, Sichuan University, 610041 Sichuan, China (http://www.scu.edu.cn/);

*Correspondence author. Address: Department of Pharmacology, School of Pharmaceutical Sciences, South-Central University for Nationalities, No. 182, Minyuan Road, 430074 Wuhan, China. Tel/Fax: +86 27 6784 2332. E-mail address: [hbtang2006@mail.scuec.edu.cn](mailto:hbtang2006@mail.scuec.edu.cn) (H. -B. Tang).

^#^G.H.T. and S.S.T. contributed equally to this work.

**Supplemental Figure and its legend**

**
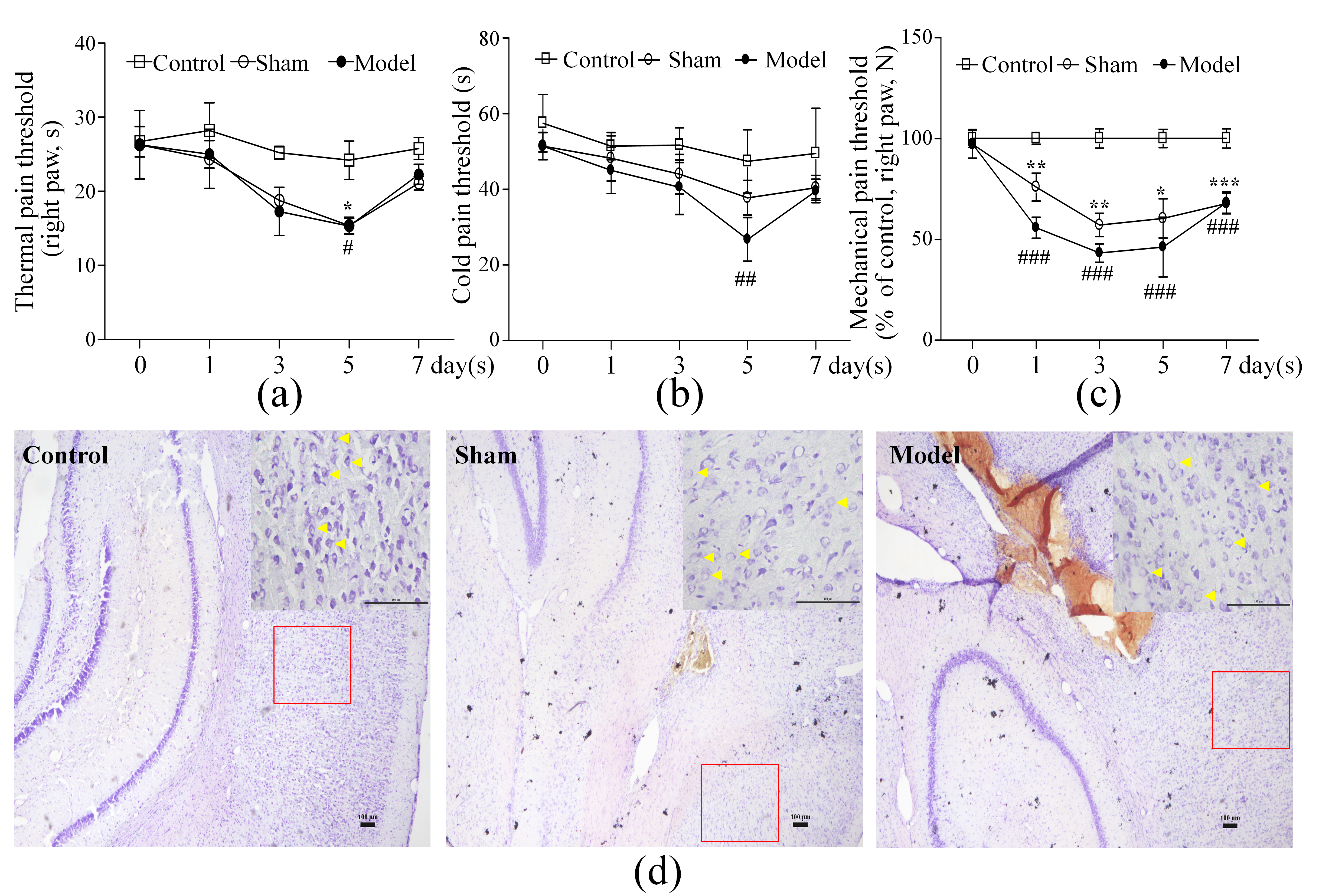
**

FIGURE I. Measurement of brain neuropathological changes and pain-related behavioral responses in CPSP rats. Changes of thermal (A), cold (B) and mechanical (C) hyperalgesia within 7 days in the contralateral paw of CPSP rats. (D) Representative photomicrographs of brain nissl staining in CPSP rats (half amount of rats in each group) on the 5^th^ day after the operation. Yellow arrows denote neuronal cells of the brain sections. Nissl-stained normal neuronal cell bodies exhibit a bright violet colour. *,**,*** denote *p* < 0.05, 0.01, 0.001 versus the control, and #, ##, ### denote *p* < 0.05, 0.01, 0.001 versus the model (n = 5 in each group;two-way analysis of variance, followed by Bonferroni *post hoc* test), respectively.
